# A multi-theory model based analysis of correlates for initiating and sustaining mammography screening behavior among Hispanic American women in the United States

**DOI:** 10.34172/hpp.2022.14

**Published:** 2022-05-29

**Authors:** Manoj Sharma, Kavita Batra, Amanda H. Wilkerson, Francesco Chirico, Siddharth Raich

**Affiliations:** ^1^Department of Social & Behavioral Health, School of Public Health, University of Nevada, Las Vegas, USA; ^2^UNLV Medicine Trauma and Critical Care, Department of Medical Education, Kirk Kerkorian School of Medicine at UNLV, University of Nevada, Las Vegas, USA; ^3^Department of Health Science, The University of Alabama, Alabama, USA; ^4^Università Cattolica del Sacro Cuore, Post-Graduate Specialization, Rome, Italy

**Keywords:** Mammography, Health behaviors, Hispanic American, Minority Breast cancer, Screening

## Abstract

**Background:** Despite the known advantages of mammography, screening rates among Hispanic American women are lower compared to other ethnic groups. Therefore, this cross-sectional study aimed to explore correlates of mammography screening behavior among a sample of Hispanic women aged 45-54 years living in the United States using the multi-theory model (MTM).

**Methods:** A 50-item web-based survey consisting of psychometrically valid tools based on MTM theoretical framework was administered through non-random sampling procedures using Qualtrics. Univariate, bivariate, and multivariate statistics were used to analyze the data.

**Results:** Out of 370 participants, nearly 49% (n=189) reported not having a mammogram in the past two years. The mean age of the sample was 48.8±2.8 years. A greater proportion of participants who have had a mammogram reported having health insurance compared to those who have not had a mammogram (93.1% vs. 75.7%, *P* <0.001). Results of hierarchical regression suggest that all MTM constructs, including participatory dialogue, behavioral confidence, and changes in the physical environment explained 33.4% of variance in initiating mammography behavior among those who have not had a mammogram. Similarly, practice for change, emotional transformation, and changes in the social environment explained 53% of the variance in sustenance of the behavior change.

**Conclusion:** Along with the MTM subscales, this study points to the important correlates such as health insurance and messaging by healthcare providers to promote the mammography seeking behavior among Hispanic women.

## Introduction

Breast cancer is one of the deadliest cancers among women worldwide.^1^ It is projected that as life expectancy continues to increase globally, new diagnoses and deaths from breast cancer will also continue to increase.^2^ In the United States, female breast cancer is the most common non-skin cancer among women, where it is projected that about 13% of American women will be diagnosed with breast cancer sometime during their lifetime.^3^ The Surveillance, Epidemiology, and End Results (SEER) data estimate 281 550 new cases of female breast cancer will be diagnosed in 2021, comprising 14.8% of all new cancer cases in the United States.^4^ Incidence and mortality from female breast cancer vary by racial and ethnic groups in the US Female breast cancer is most common among older, middle-aged women, as well as white women (129.1 new cases per 100 000) when compared to both Black (124.7 new cases per 100 000) and Hispanic women (100.3 new cases per 100 000).^4^

Preventive screening for breast cancer, particularly mammography, helps to facilitate early cancer diagnosis and prompt treatment. The United States Preventative Services Task Force (USPSTF) recommends women who are 50 to 74 years of age and at average risk for breast cancer receive a mammogram every two years^5^; whereas, the American Cancer Society recommends women age 45 to 54 at average risk receive a mammogram annually, followed by a reduction to biannual screenings at age 55 and older.^6^ This study was confined to the ages 45-54 years and used the conservative guideline of getting mammograms every year for this age group. Recent research has documented that regular participation in mammography screenings reduces breast cancer mortality, particularly among women who participate in at least two consecutive mammogram screening examinations.^7^ Despite the advantages of mammography screening, screening rates in the US vary substantially by racial and ethnic group. Although Hispanic women have a lower incidence of breast cancer when compared to non-Hispanic white and Black women, it is well documented that Hispanic women have consistently lower mammography screening rates when compared to their non-Hispanic counterparts.^8-12^

Lower mammography screening rates among Hispanic women in the United States are likely caused by a multitude of factors, including intrapersonal factors such as fear of cancer diagnosis, fear of negative appearance (if intervened by mastectomy), fear of pain, and lower awareness of cancer-related risk factors and cancer screening requirements as well as socioeconomic barriers, including lack of health insurance, perceived discrimination, lower socioeconomic status, lower education, and higher unemployment rates.^8-12^ Further, research suggests that culture-specific issues and barriers, such as fear of cancer, embarrassment, fatalistic perspectives, language barriers, and low perceived susceptibility, are also associated with lower mammography screening rates in Hispanic women.^13^ However, there are inconsistencies in factors associated with lower mammography screening rates in Hispanic women, supporting the need for further research to explore additional factors and theoretical frameworks to explain low mammography rates in this sub-group of women in the United States.

The present study utilized a fourth-generation contemporary health behavior theoretical framework, the multi-theory model (MTM) to explore additional factors associated with mammography rates among a sample of Hispanic women in the United States. The MTM is a theoretical framework used to explain both the initiation and sustenance of behavior change.^14^ The MTM is adaptable across a wide variety of health behaviors and has been used to explain a multitude of health behaviors in varying populations,^15-18^ including other preventative health behaviors such as HPV vaccination intention^19^ and COVID-19 vaccination intention.^20^

The utilization of robust theoretical frameworks, such as the MTM, allows researchers and practitioners to identify and better understand factors associated with intentions to receive mammography screenings among Hispanic women in the United States. The identification of factors associated with not only the initiation but also the sustenance of mammography screening behavior allows researchers and practitioners to design effective, theory-based intervention strategies to increase mammography screening behavior in this demographic sub-group.^21^ Therefore, the purpose of this study was to determine if the MTM could explain initiation and sustenance of mammography screening behavior among a sample of Hispanic women in the United States to inform theory-based recommendations for health promotion interventions to increase mammography screening behavior in Hispanic women.

## Materials and Methods

### Study design, participants, and sampling

This nationwide study was cross-sectional and descriptive in nature with an aim to explain the correlates of mammography screening behavior among Hispanic American women aged 45-54 years. Participants who were able to understand English and provided voluntary consent were included in this study. Data for this study were collected in October 2021(10/3/2021-10/31/2021). Data collection efforts were performed by the Qualtrics Research Marketing team as a part of the contractual agreement with the study investigators. Commercial sampling differs in many ways from traditional sampling, and the differences have been described by previous studies.^22^ With regards to Qualtrics methodology in recruiting a desired and high-quality sample, multiple avenues of data collection including recruitment through apps, games, and social media are utilized. Efforts were made to recruit a representative sample in terms of regional distribution through quota sampling. At the beginning of the survey, a few screening questions (without the disclosure of the research objectives) were introduced to minimize self-selection and response bias. Potential participants were eligible to take the survey if they self-identified as a Hispanic American woman living in the United States. through the screening questions. Due to the use of multiple sources or panels for sampling, the response rate was not computed. Eligible participants who completed the survey were given incentives per terms and conditions set forth by Qualtrics and its data collection partners.

### Quality control and data management

Qualtrics Research Marketing Team provides quality control and data management services during data collection. For this study, Qualtrics sent out survey invitations to recruit an initial sample and provided “soft launch data” to the authors, which helped to catch early data idiosyncrasies and to ensure a good quality check. In addition, Qualtrics performed data scrubbing to remove low-quality responses. Responses which took less than half of the median length of survey completion were considered inferior quality responses or flagged as speeders.^23,24^ For instance, in the current survey, the median length of completion was 388 seconds (6.46 minutes), therefore participants (n = 18) who took less than 194 seconds (3.23 minutes) to complete the survey were excluded. Options in Qualtrics such as digital fingerprinting and “prevent ballot box stuffing” were used to limit one response from each participant. The survey distribution was completely anonymous, which means no identifiers were collected through this survey. Data were secured in a password protected file, which was housed in a password protected computer device.

### Survey tool and measures

**Figure 1:**
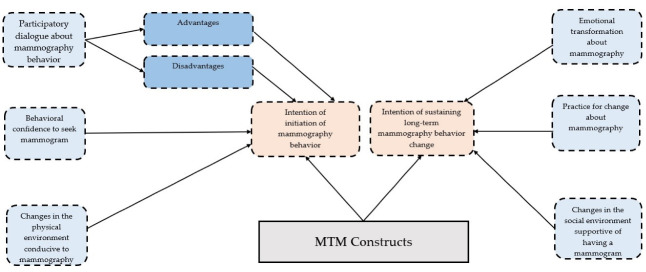
The framework of Multi Theory Model of mammography behavior

A 50-item questionnaire containing 13 demographic questions, 31 questions from a previously validated MTM tool^25^ and 6 additional items from the Fear of Negative Appearance Evaluation scale (FNAES)^26,27^ was used in the present study. The MTM tool is based on the fourth-generation behavioral theory, MTM, and is known for its well-established psychometric properties observed while assessing mammography behavior in a sample of Asian American women.^26,27^ The MTM questionnaire (Figure 1) measures initiation and sustenance of behavior change. For the initiation model, there are three subscales namely, participatory dialogue (measured through perceived advantages and perceived disadvantages), behavioral confidence and changes in the physical environment.^27^ Perceived advantages are defined as the perception of the positive consequences resulting from adopting a certain behavior or action, which is undergoing mammography in the current study. In contrast, perceived disadvantages refer to the perception of the negative consequences. Both perceived advantages and disadvantages are measured through 5 items each on a 5-point Likert scale ranging from “strongly disagree” (0) to “strongly agree” (4) with a maximum possible score of 20 units.

The difference derivative of advantages and disadvantages is termed as participatory dialogue, which has a score range of –20 to +20 units. Positive scores are indicative of behavioral change. Next, behavioral confidence (i.e. sure expectation in adopting a particular behavior) and changes in physical environment (i.e. enabling physical environment factors to initiate behaviors) were measured using five and three items, respectively. The construct behavioral confidence relates to an individual’s sureness in their ability to engage in the behavior change that can emanate from self or external sources such as belief in a deity, belief in a powerful other, belief in the Almighty, etc. The possible score of behavioral confidence is 0-20 points while for changes in the physical environment, the score ranged from 0-12 units. The 5-item Likert scale with end points of “not at all sure (0)” to “completely sure (4)” was used. Higher score values of perceived advantages, perceived disadvantages, behavioral confidence, and changes in the physical environment indicate greater likelihood of initiating a behavior. For sustenance, there were three subscales, namely emotional transformation, practice for change, and changes in the social environment. Emotional transformation is defined as the ability to turn negative emotional state to the positive one. Practice of change denotes ability to sustain a behavior even in the presence of challenges, and “changes in social environment” is leveraging social support to sustain a behavior. Aside from changes in the social environment, all other subscales were measured using three items. A 5-item Likert scale with end points of “not at all sure (0)” to “completely sure (4)” was used.

### A-priori power analysis and sample size justification

Using the G*Power 3.1.9.7 software (linear multiple regression: fixed model, R^2^ increase), a minimal number of 154 participants was required to reach significance when considering the following statistical parameters: type I error α = 5%, power 1-β = 95%, a moderate effect size f^2^ = 0.15, and a total number of variables N = 10 to be integrated in the multivariable regression analysis.^28,29^ For a medium effect size and aforementioned statistical parameters, the minimum sample size for independent samples t-test and chi-square analysis were 210 and 220 respectively. We took the highest value as the potential sample size (N = 220) as this fulfilled the minimum sample requirements to investigate hypothesized effects and added 10% to account for missing data,^29,30^ resulting in a target sample size of 242 participants.

### Analyses methods

SPSS software v.26 (Armonk, NY, USA: IBM Corp.) was used to analyze the data. All types of analytic methods, including univariate, bivariate, and multivariate statistics were utilized. First, univariate statistics were calculated to describe the characteristics of the sample. As an uncertainty measure, 95% confidence intervals of proportion were calculated through normal approximation to the binomial distribution. Initial model and assumptions (e.g. independence of residuals, linearity, equal error variance, multicollinearity, and normality of residuals) were tested prior to the predictive modelling. Comparisons across categorical and continuous outcomes were derived through Chi-square and independent sample t-tests respectively (bivariate tests). Pearson correlation was utilized to investigate bivariate relationships between the observed variables. Two separate (one for initiation and one for sustenance) hierarchical regression models were used to explain the change in variance in the dependent variables (initiation and sustenance of mammography screening) attributed to the sequential addition of independent variables. Dummy coding was applied to the polytomous variables for the accurate calculation of regression slopes and coefficients. A detailed model building process can be seen in Table 1. The statistical significance was set a priori at *P* < 0.05.

**Table 1:** Model building algorithm for the Hierarchical) Regression Analysis

**Initiation as a dependent variable**	
Model 1	Initiation = Intercept + Age + Residence + Employment+ Insurance
Model 2	Initiation = Intercept + Model 1 variables + Participatory dialogue
Model 3	Initiation = Intercept + Model 2 variables + Behavioral confidence
Model 4	Initiation = Intercept + Model 3 variables + Changes in the physical environment
**Sustenance as dependent variable**	
Model 1	Sustenance = Intercept + Age + Residence + Employment + Insurance
Model 2	Sustenance = Intercept + Model 1 variables + Emotional transformation
Model 3	Sustenance = Intercept + Model 2 variables + practice for change
Model 4	Sustenance = Intercept + Model 3 variables + changes in the social environment

## Results

### Univariate statistics 

A total of 370 valid responses were analyzed, exceeding the required sample size in the power analysis. In the study sample, a comparable proportion of participants have and have not had mammography over the past year (51.1% vs. 48.9%, Table 2). Among those who have had mammography, nearly 89% reported having a normal mammogram. The mean age of the sample was 48.8 ± 2.8 years, and the average duration of U.S. residency status was 40.6 years (SD = 14.2 years). A vast majority of the participants identified as Mexican followed by Puerto Rican. The participant distribution was comparable among suburban and urban residential areas. A large proportion of the participants were married (44.6%) and had religious affiliation of being Roman Catholic (46.5%). The regional distribution of the sample was consistent with the census distribution^31^ population parameters with 39.2% participants were from Southern region of the country.

**Table 2:** Univariate demographic statistics of the study population (N = 370)

**Variable**	**Categories**	**No. (%)**	**95% CI (LCL, UCL)**
Had mammography over the past year	Yes	189 (51.1)	45.9, 56.3
	No	181 (48.9)	43.7, 54.1
Age in years (mean ± standard deviation)		48.8 ± 2.8	48.5, 49.1
Hispanic ethnic subgroups	Mexican	204 (55.1)	49.9, 60.3
	Puerto Rican	76 (20.5)	16.5, 25.0
	Cuban	18 (4.9)	2.9, 7.6
	Other	68 (18.4)	14.6, 22.7
Religion	Roman Catholic	172 (46.5)	41.3, 51.7
	Protestant	34 (9.2)	6.5, 12.6
	Nothing in particular	49 (13.2)	9.9, 17.1
	Other	115 (31.1)	26.4, 36.1
Marital status	Married	165 (44.6)	39.5, 49.8
	Never married	58 (15.7)	12.1, 19.8
	Divorced/Separated	100 (27.0)	22.6, 31.9
	Other	47 (12.7)	9.5, 16.5
Residence	Rural	81 (21.9)	17.8, 26.5
	Suburban	143 (38.6)	33.6, 43.8
	Urban	146 (39.5)	34.5, 44.5
Region	Midwest	62 (16.8)	13.1, 21.0
	Northeast	69 (18.6)	14.8, 23.0
	South	145 (39.2)	34.2, 44.4
	West	94 (25.4)	21.1, 30.2
Health insurance	Yes	313 (84.6)	80.5, 88.1
	No	57 (15.4)	11.8, 19.5
Employed	Yes	220 (59.5)	54.3, 64.5
	No	150 (40.5)	35.5, 45.7

Note: Some percentages may not add up to 100% as a few participants preferred not to answer. CL: Confidence interval; LCL: lower confidence level; UCL: upper confidence level.

### Bivariate statistics

Results of bivariate analysis revealed statistically significant differences in healthcare access characteristics among participants who have (group 1) and who have not had mammography (group 2) over the past year (Table 3). In group 1, a greater proportion of participants reported having health insurance compared to the group 2 (93.1% vs. 75.7%, *P* < 0.001, Table 3). No statistically significant differences in proportions were found by education, employment status, and annual gross income. In addition, age of these two groups was not statistically significant for group 1 and group 2 (48.8 ± 2.8 vs. 48.6 ± 2.6 years; *P* = 0.212). Among those who have had mammogram, nearly 93% of respondents reported having it prescribed by their healthcare providers compared to only 49.2% among those who have not had the mammogram (*P* < 0.001, Table 3).

**Table 3:** Bivariate analysis for comparing socio-economic and healthcare access characteristics of the groups who have and have not had mammography over the past year (N = 370)

**Variable**	**Categories**	**Participants who have had mammography** **No. (%)**	**Participants who have not had mammography** **No. (%)**	**P value**
Total sample	Group sizes	189 (51.1)	181 (48.9)	
Education	Less than high school diploma	7 (3.7)	15 (8.3)	0.061
	High school graduate	32 (16.9)	40 (22.1)	
	Associate degree	32 (16.9)	33 (18.1)	
	Bachelor degree	41 (21.7)	26 (14.4)	
	Some college but no degree	48 (25.4)	45 (24.9)	
	Trade school	8 (4.2)	12 (6.6)	
	Graduate degree	21 (11.1)	10 (5.5)	
Healthcare insurance	Yes	176 (93.1)	137 (75.7)	< 0.001*
	No	13 (6.9)	44 (24.3)	
Employed	Yes	116 (61.4)	104 (57.5)	0.4
	No	73 (38.6)	77 (42.5)	
Income	< $25,000	44 (23.3)	55 (30.4)	0.6
	$25,000-$50,000	56 (29.6)	59 (32.6)	
	$50,001-$75,000	48 (25.4)	36 (19.9)	
	$75,001-$100,000	20 (10.6)	15 (8.3)	
	$100,001-$125,000	6 (3.2)	5 (2.8)	
	$125,001-$150,000	9 (4.8)	5 (2.8)	
	> $150,001	6 (3.2)	6 (3.3)	
Prescribed mammography by healthcare provider	Yes	175 (92.6)	89 (49.2)	< 0.001*
	No	14 (7.4)	92 (50.8)	

*P values < 0.05 are considered statistically significant.

**Figure 2:**
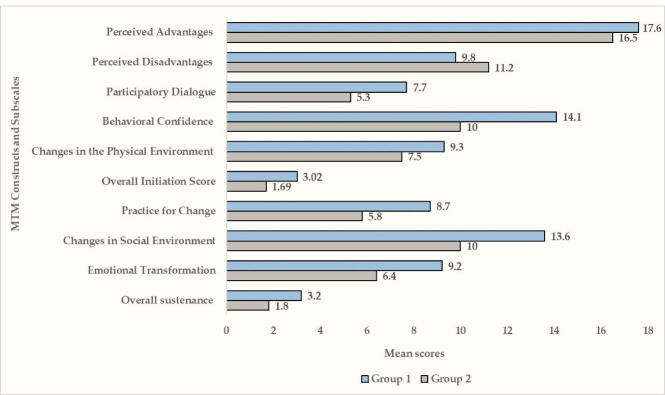
Comparing mean scores of MTM constructs among participants who have (group 1) and who have not had mammography (group 2) over the past year

Upon deriving group-wise comparisons of MTM constructs and subscales, the mean scores of perceived advantages (17.6 ± 2.8 vs. 16.5 ± 3.2), participatory dialogue (7.7 ± 4.6 vs. 5.3 ± 4.8, behavioral confidence (14.1 ± 4.5 vs. 10.0 ± 4.8), changes in physical environment (9.3 ± 2.7 vs. 7.5 ± 3.4) and overall initiation (3.02 ± 0.99 vs. 1.69 ± 1.41) were statistically significantly higher among group 1 compared to group 2 (*P* < 0.001; Figure 2). In contrast, the mean score of perceived disadvantages was higher among those who have not had the mammogram (group 2) compared to those who have had a mammogram (group 1). Similar to the overall initiation subscales, sustenance subscales had a higher mean among group 1 compared to group 2 (*P* < 0.001). The mean scores of subscales: emotional transformation (9.2 ± 2.6 vs. 6.4 ± 3.4), practice for change (8.7 ± 2.7 vs. 5.8 ± 3.3), changes in social environment (13.6 ± 4.5 vs. 10.0 ± 5.1), and overall sustenance (3.2 ± 0.9 vs. 1.8 ± 1.3) were significantly higher among group 1 as compared to the group 2 (Figure 2).

Table 4 indicates the Pearson correlation coefficient matrix of all MTM subscales and the Fear of Appearance scale. Perceived advantages were indirectly correlated with perceived disadvantages (r = -0.12, *P* < 0.01), directly correlated with behavioral confidence (r = 0.24, *P* < 0.01), changes in physical environment (r = 0.30, *P* < 0.01), emotional transformation (r = 0.40, *P* < 0.01), practice for change (r = 0.35, *P* < 0.01), and changes in social environment (r = 0.44, *P* < 0.01). Perceived disadvantages were indirectly correlated with behavioral confidence (r = -0.26, *P* < 0.01), changes in the physical environment (r = -0.22, *P* < 0.01), emotional transformation (r = -0.27, *P* < 0.01), practice for change (r = -0.22, *P* < 0.01), changes in the social environment (r = -0.18, *P* < 0.01), and directly correlated with the fear of appearance (r = 0.26, *P* < 0.01). Changes in physical environment was indirectly correlated with the fear of appearance (r = -0.12, *P* < 0.05) and directly correlated with the behavioral confidence (r = 0.71, *P* < 0.01). There was a strong and direct correlation observed between emotional transformation and practice for change (r = 0.80, *P* < 0.01). Changes in physical and social environment were moderately and directly correlated (r = 0.55, *P* < 0.01). The MTM instrument and fear of appearance scale showed good internal consistency reliability. The MTM subscales had Cronbach alpha values ranging from 0.73-0.91 (Table 4), and the Cronbach’s alpha value for the fear of appearance scale was 0.95.

**Table 4:** Bivariate correlations, and reliability diagnostics for MTM variables and Fear of Appearance (N = 370)

**Variable**s	**1**	**2**	**3**	**4**	**5**	**6**	**7**	**8**
1. Advantages	1	-0.12*	0.24**	0.30**	0.40**	0.35**	0.44**	-0.04
2. Disadvantages	-0.120*	1	-0.26**	-0.22**	-0.27**	-0.22**	-0.18**	0.26**
3. Behavioral Confidence	0.24**	-0.26**	1	0.71**	0.63**	0.69**	0.54**	-0.10*
4. Changes in the Physical Environment	0.30**	-0.22**	0.71**	1	0.64**	0.67**	0.55**	-.12*
5. Emotional Transformation	0.40**	-0.27**	0.63**	0.64**	1	0.80**	0.64**	-0.09
6. Practice for Change	0.35**	-0.22**	0.69**	0.67**	0.80**	1	0.70**	-0.05
7. Changes in Social Environment	0.44**	-0.18**	0.54**	0.55**	0.64**	0.70**	1	-0.05
8. Fear of Appearance	-0.04	0.26**	-0.10*	-0.12*	-0.098	-0.05	-0.05	1
Cronbach alpha values	0.81	0.73	0.88	0.88	0.90	0.91	0.86	0.95

*Significant below 0.05; ** Significant below 0.01.

### Comparison of groups through the fear of negative appearance scale

**Figure 3:**
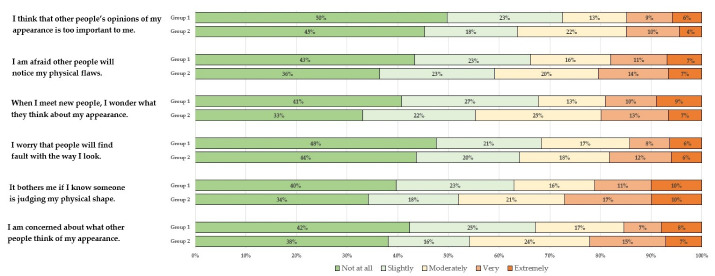
Item-wise analysis of "Fear of Appearance Scale" among participants who have (group 1) and who have not had mammography (group 2) over the past year

Upon analyzing all the items related to the “Fear of Negative Appearance” scale, it was found that higher proportion of participants in the group 1 were “not at all” concerned about other people’s opinions or judgements about their physical appearance compared to the participants in the group 2 (Figure 3).

### Hierarchical regression

In a hierarchical regression model for initiation, the fourth model (i.e., final model) explained nearly 33.4% of variance in initiating mammography behavior among participants (n = 189) who have not had a mammogram over the past year (adjusted R^2^ = 0.334, F = 12.294, *P* < 0.001, Table 5). With each unit increment in subscales of initiation (i.e. participatory dialogue, behavior confidence and changes in physical environment), the conditional mean for initiating mammography behavior increased by 0.038, 0.090, and 0.066 units, respectively (Model 4, Table 5). None of the slopes of demographic and healthcare access variables were significant, which indicated no significant differences in the conditional mean changes in initiating mammography uptake behavior among participants who have not had mammography over the past year. In a multilevel regression model of sustenance, the fourth model (i.e., final model) explained nearly 53% of variance in the sustenance of mammography behavior among participants (n = 181) who have not had a mammogram in the past year (adjusted R^2^ = 0.527, F = 18.329, *P* < 0.001, Table 5). With each unit increment in the sustenance subscales (i.e. practice for change and changes in social environment), the conditional mean for sustaining mammography uptake behavior increased by 0.173 and 0.076 respectively (Model 4, Table 5). The slopes of demographic, healthcare access, and emotional transformation were not significant, which indicated no significant differences in the conditional mean changes in the sustenance of mammography uptake behavior among participants who have not had a mammogram in the past year.

**Table 5:** Hierarchical regression to predict likelihood for initiation and sustenance of mammography behavior among participants who have not had the mammogram over the past 1 years (n = 181)

**Variable**s	**Model 1**		**Model 2**		**Model 3**		**Model 4**	
	**B**	**β**	**B**	**β**	**B**	**β**	**B**	**β**
**Initiation as a dependent variable**								
Constant	1.293	-	1.247	-	0.860	-	0.733	-
Age	0.018	0.038	0.012	0.025	-0.004	-0.008	-0.006	-0.018
Residence (Ref: Urban)								
Suburban	-0.208	-0.081	-0.262	-0.102	-0.160	-0.062	-0.190	-0.074
Rural	0.112	0.040	0.208	0.073	0.212	0.075	0.192	0.067
Health insurance (Ref: Yes)	-0.627	-0.214*	-0.720	-0.246**	-0.446	-0.152*	-0.368	-0.126
Employment status (Ref: Yes)	0.120	0.047	0.065	0.025	0.051	0.020	0.122	0.048
Participatory dialogue		-	0.073	0.339**	0.044	0.206*	0.038	0.177*
Behavioral confidence		-	-	-	0.119	0.460**	0.090	0.351**
Changes in the physical environment		-	-	-	-	-	0.066	0.181*
R^2^	0.050	-	0.162	-	0.348	-	0.364	-
F	1.836	-	5.607**		13.181**	-	12.294**	-
Δ R^2^	0.050	-	0.112	-	0.186	-	0.016	-
Δ F	1.836	-	23.290**	-	49.289**	-	4.319*	-
**Sustenance as a dependent variable**								
Constant	0.094	-	0.014	-	-0.111	-	-1.202	-
Age	0.038	0.078	0.005	0.011	0.004	0.008	0.021	0.044
Residence (Ref: Urban)								
Suburban	-0.104	-0.039	0.074	0.027	0.033	0.012	0.023	0.009
Rural	0.050	0.017	0.127	0.043	0.007	0.002	-0.011	-0.004
Health insurance (Ref: Yes)	-0.419	-0.137	-0.095	-0.031	0.020	0.007	0.058	0.019
Employment status (Ref: Yes)	0.087	0.033	0.083	0.032	0.063	0.024	0.024	0.009
Emotional transformation	-	-	0.235	0.614**	0.074	0.193*	0.036	0.094
Practice for change	-	-	-	-	0.217	0.546**	0.173	0.435**
Changes in the social environment	-	-	-	-	-	-	0.076	0.297**
R^2^	0.026	-	0.385	-	0.500	-	0.548	-
F	0.922	-	18.170**	-	24.751**	-	26.117**	-
Δ R^2^	0.026	-	0.360	-	0.115	-	0.048	-
Δ F	0.922	-	101.758**	-	39.877**	-	18.329**	-

* *P*value < 0.05; ** *P* value < 0.001; Adjusted R^2^initiation = 0.334; Adjusted R^2^sustenance = 0.527

## Discussion

The study aimed to identify determinants of mammography screening in a national sample of Hispanic American women based on the constructs of the MTM of health behavior change and salient demographic characteristics. As supported by previous studies,^8-12^ this study also found that Hispanic American women had substantially lower mammography screening rates, with only 51.1% of participants reporting having a mammogram in the last year preceding the survey. This finding confirms the need to target this subsection of the population for concerted mammography promotion campaigns and interventions.

In comparing demographic variables between those who had a mammogram and those who did not have mammogram, two variables emerged as having a significant difference between the two groups, namely having health insurance and having a recommendation by a healthcare provider, both being higher in the former. This finding was expected and in consonance with the existing literature.^9,10^ The finding points to the important role that having health insurance and recommendation from healthcare providers can have in the decision to have a mammogram among Hispanic American women. There is an urgent need to educate primary care physicians and women’s health practitioners (i.e., obstetricians, gynecologists, and women’s health nurse practitioners) to emphasize the need to get mammograms at regular intervals for their patients, especially those who identify as Hispanic American. Signage in Spanish and using culturally appropriate imaging and language at clinics and primary health centers reinforcing such messages can also go a long way to promote mammography. Additionally, workplace health promotion programs, including screening programs may be beneficial to raise the awareness among employed females. A coordinated approach between public and occupational health stakeholders will be critical in overcoming barriers in the physical environment.^32^

In the initiation of mammography, among those Hispanic American women who had not had mammograms over the past year, all the three MTM constructs of participatory dialogue, behavioral confidence, and changes in the physical environment were significant predictors and accounted for 33.4% of the variance in initiation of a mammogram in the next year. This is a substantial proportion of the variance to be explained in a health behavior research study.^27^ This finding is also supported by a similar study about mammography and the role of MTM conducted with Asian American women that found that all three constructs of MTM were significant predictors.^25^ Besides lending support to MTM, the finding emphasizes the role of several constructs to increase mammography screening for Hispanic American women, such as emphasizing the pros over cons of getting mammograms done, building surety in women to get the procedure done, and providing environmental supports in the form of measures such as health insurance, proximity to clinics, and transportation. The construct of participatory dialogue that underscores the pros over cons of getting mammograms can be facilitated by a healthcare provider or health educator in an open way to address apprehensions in the minds of women who are hesitant about mammography screening. The construct of behavioral confidence that builds surety in women to get the procedure done can be facilitated by health professionals through exploring sources of such confidence which can be from sources such as self, deity, powerful other, Almighty, etc. to build faith in their ability to get the procedure done. The construct of changes in the physical environment can be facilitated by providing tangible supports to women who experience physical barriers to accessing mammography.

With regard to regular maintenance of getting mammograms every year by Hispanic American women, the study found that all three constructs of the sustenance model in the MTM, emotional transformation, practice for change, and changes in the social environment, were statistically significant predictors and accounted for a substantial 52.7% of the variance in the behavior.^27^ A similar study done with Asian American women found that emotional transformation and practice for change were significant MTM predictors for sustaining regularity in getting mammograms and accounted for a similar 53.9% explanation in the variance.^25^ The fact that changes in the social environment was a significant factor for Hispanic American women points to the importance that social influences such as family and friends play in the decision-making in this sub-cultural group. Measures to have messaging for and through social influences need to be incorporated in the mammography promotion programs and campaigns for Hispanic American women. Healthcare providers and health educators must help women self-identify their emotions or feelings and direct those toward goal setting and self-motivation in the form of getting regular mammograms. Regarding the construct of practice for change, strategies such as reflection, monitoring, and learning about overcoming barriers are some strategies that health professionals and health educators can teach their clients to sustain regular mammogram screenings in the long-term.

The study also looked at the construct of fear of negative appearance and found that a higher percentage of respondents who had mammograms in the past year were less concerned about other people’s opinions or judgments about their physical appearance compared to those who did not have mammograms in the past year. Fear of negative appearance has previously not been studied in the context of getting mammograms but only studied with eating behaviors.^26^This is a new contribution to the literature in this regard. From the perspective of designing educational interventions for promoting mammograms, this finding can be used to support programs that reduce participants’ undue obsession with their appearance and address any appearance-based apprehensions women may associate with mammography.

### Strengths and limitations of the study

This study was among the few theory-based studies that have been conducted to understand mammography screening behavior and the first to study this behavior among Hispanic American women utilizing a contemporary fourth-generation theoretical framework, the MTM. The study also provided support to add to the growing evidence in the literature for the utility of the MTM, which is also a strength of the study. However, the study was not without limitations. The study relied on self-reported data. While it is indispensable to use this form of data collection to study theoretical antecedents of behavior change (i.e., attitudes, beliefs), the behavior of getting mammograms could have been reported from clinical charts or other objective means. However, the cost and practical issues prevented such data collection. Self-reported data are subject to biases, such as social desirability and recall bias, that must be kept in mind while interpreting the findings from this study. For instance, information related to respondent’s residence (rural/urban/suburban) was self-reported, which might have led to the information bias. The study also utilized a cross-sectional design from which causal inferences cannot be made, as all the variables are being recorded at the same point in time. Since the participants were given incentives, the sample was not random, which may restrict the generalizability of the results. However, for model testing, such samples are deemed appropriate.^27^ Finally, the study was conducted during the COVID-19 pandemic, which might have impacted the rate of mammography utilization. Previous studies reported a cumulative deficit in the mammography utilization as compared to the pre-pandemic volumes, particularly among Hispanic women.^33^

## Conclusion

The study found that there is a need to enhance health insurance coverage and messaging by healthcare providers to deliver concerted messages to Hispanic American women regarding receiving regular mammograms. This behavior can be initiated through initiating dialogue that underscores advantages over disadvantages, building confidence in the patient’s ability to get a mammogram, and providing necessary tangible supports. The behavior of getting regular mammograms among Hispanic American women can be maintained through converting emotions into self-motivational goals, monitoring the behavior, overcoming barriers, and using social support. Overall, the MTM holds promise to improve health promotion programming among Hispanic American women to promote both the initiation and continuation of mammogram screenings for the early detection of breast cancer. Tailored intervention, particularly that employ the fourth- generation theoretical model, such as MTM will be critical to understand determinants of mammography behavior to encourage women to undergo mammography.

## Acknowledgements

Authors would like to thank their respective institutions for their kind support.

## Authors’ contributions

Conceptualization, M.S. and K.B.; Methodology, M.S. and K.B.; Software, K.B.; Validation, K.B., and M.S.; Formal Analysis, K.B.; Investigation, K.B., M.S., A.H.W., F.C.; Resources, M.S.; Data Curation, K.B.; Writing – Original Draft Preparation, K.B., M.S., A.H.W., F.C.; Writing – Review & Editing, K.B., M.S., A.H.W., F.C., S.R.; Visualization, K.B.; Supervision, M.S.; Project Administration, M.S., K.B.; Funding Acquisition, M.S.

## Funding

This research was funded by the School of Public Health, University of Nevada, Las Vegas internal grant number PG03008. This research received no external funding.

## Ethical approval

This study (protocol ID: 1800066-1 dated August 31, 2021) received an exempt status from the Institutional Review Board at the University of Nevada, Las Vegas. All research activities were performed in accordance with the Declaration of Helsinki. Informed consent was obtained from all participants.

## Competing interests

None.
